# Brain gray and white matter abnormalities in preterm-born adolescents: A meta-analysis of voxel-based morphometry studies

**DOI:** 10.1371/journal.pone.0203498

**Published:** 2018-10-10

**Authors:** Le Zhou, Youjin Zhao, Xinghui Liu, Weihong Kuang, Hongyan Zhu, Jing Dai, Manxi He, Su Lui, Graham J. Kemp, Qiyong Gong

**Affiliations:** 1 Huaxi MR Research Center (HMRRC), Department of Radiology, West China Hospital of Sichuan University, Chengdu, Sichuan, P.R. China; 2 Health Management Department, West China Hospital of Sichuan University, Chengdu, Sichuan, P.R. China; 3 Obstetrics and Gynecology Department, West China Second University Hospital, Sichuan University, Chengdu, Sichuan, P.R. China; 4 Department of Psychiatry, West China Hospital of Sichuan University, Chengdu, Sichuan, P.R. China; 5 Laboratory of Stem Cell Biology, State Key Laboratory of Biotherapy, West China Hospital of Sichuan University, Chengdu, Sichuan, P.R. China; 6 Department of Psychoradiology, Chengdu Mental Health Center, Chengdu, Sichuan, P.R. China; 7 Liverpool Magnetic Resonance and Imaging Centre (LiMRIC) and Institute of Ageing and Chronic Disease, University of Liverpool, Liverpool, United Kingdom; Nagoya Mental Clinic, JAPAN

## Abstract

**Introduction:**

Studies using voxel-based morphometry report variable and inconsistent abnormalities of gray matter volume (GMV) and white matter volume (WMV) in brains of preterm-born adolescents (PBA). In such circumstances a meta-analysis can help identify the most prominent and consistent abnormalities.

**Method:**

We identified 9 eligible studies by systematic search of the literature up to October 2017. We used Seed-based d Mapping to analyze GMV and WMV alterations between PBA and healthy controls.

**Results:**

In the GMV meta-analysis, PBA compared to healthy controls showed: increased GMV in left cuneus cortex, left superior frontal gyrus, and right anterior cingulate cortex; decreased GMV in bilateral inferior temporal gyrus (ITG), left superior frontal gyrus, and right caudate nucleus. In the WMV meta-analysis, PBA showed: increased WMV in right fusiform gyrus and precuneus; decreased WMV in bilateral ITG, and right inferior frontal gyrus. In meta-regression analysis, the percentage of male PBA negatively correlated with decreased GMV of bilateral ITG.

**Interpretation:**

PBA show widespread GMV and WMV alterations in the default mode network, visual recognition network, and salience network. These changes may be causally relevant to socialization difficulties and cognitive impairments. The meta-regression results perhaps reveal the structural underpinning of the cognition-related sex differences in PBA.

## Introduction

Nearly 10% of all live births worldwide are preterm (before 37 weeks of gestation)[1, 2]. Although the survival of very preterm infants has improved, preterm-born adolescents (PBA) are at risk for long-term abnormalities of neurodevelopment [1, 3, 4]. These can manifest as socialization difficulties [5], educational underachievement[6], language impairment[7], motor dysfunction[8], cognitive delay[9], and emotional/behavior adjustment problems[3] in both preschool and school-aged children born preterm [1, 6, 9, 10]. PBA are also at increased risk of developing psychiatric disorders, including schizophrenia[1], bipolar affective disorder[11], autism[10], depression and anxiety disorder[1, 4, 11]. Many studies have shown that the abnormality of cognition and behavior are in related with brain structure alterations and brain function alterations. We hypothesized that preterm birth would result in long-lasting changes in brain development. It is therefore important to understand the effects of preterm birth on the brain, particularly its long-term impact on neurodevelopment.

Over the last 20 years, magnetic resonance imaging (MRI) had been widely used to investigate brain abnormalities in psychiatric disorders. There are several MRI analytic approaches to quantifying structural abnormalities, including traditional hand-drawn regions of interest (ROIs) and whole-brain morphometrics. Voxel-based morphometry (VBM) is an automated whole-brain technique with comparable accuracy to ROI approaches. VBM compares regional GM or WM changes in terms of density and volume in all brain areas through structural MRI scans. It can detect subtle changes in the brain MRI images between groups of subjects[12].

Reported gray matter volume (GMV) and white matter volume (WMV) abnormalities in PBA have been variable and inconsistent: increased GMV in the fusiform gyrus[13]; decreased GMV in the temporal[14–16], parietal[16, 17] and prefrontal[14] cortex, and in the hippocampus[16], and caudate nucleus[15]; increased WMV in the parahippocampal region and cerebellum[18]; and decreased WMV in the temporal and frontal regions[15]. Some results are contradictory, probably owing to methodological differences and small or heterogeneous study samples: for example, Meng et al. reported increased GMV in the posterior cingulate cortices[19], whereas Lean et al. found decreased GMV in part of this region [16]. Soria-Pastor et al. did not observe any region of increased GMV in PBA[17].

Therefore, identifying prominent and consistent results from VBM studies of WMV and GMV in PBA through meta-analysis is of particular significance. This was the first aim of this study. The second aim was to examine the effects of demographics and clinical characteristics on GMV and WMV in PBA, using Seed-based d Mapping (formerly "Signed Differential Mapping") (SDM). SDM is a relatively reliable and valid quantitative coordinate-based meta-analytic tool. It has the technical advantage over other meta-analytical tools that the same map includes positive and negative findings from contributing studies. SDM has been successfully applied to neuroimaging studies of several psychiatric disorders such as anxiety disorders, bipolar disorder, Alzheimer's disease and posttraumatic stress disorder [1, 11, 20, 21]. It possesses good overlap with pooled analysis, adequate sensitivity, and excellent control over false positives[22].

In brief, we conducted separate meta-analyses of VBM studies on GMV and WMV, in order to find the long-lasting changes in brain development.

## Methods and materials

### Literature searches

Meta-analysis was conducted according to the Preferred Reporting Items for Systematic reviews and Meta-Analyses guidelines (PRISMA)[23]. A systematic search strategy was conducted using PubMed, Embase, Web of Science and Science Direct up to October 2017, with the following search terms: “premature labor” or “preterm infant” or “premature birth” or “premature delivery” or “preterm born” and “voxel-based morphometry” or “VBM” or “morphometry”. The reference lists of the articles included in the review were manually checked to identify further studies for inclusion. There was no language restriction, though all included articles were written in English.

We included studies which: (1) used VBM to analyze GMV and/or WMV changes in PBA; (2) compared PBA and healthy controls (HC); (3) performed a whole-brain analysis; and (4) reported coordinates in a defined stereotaxic space (i.e. Montreal Neurological Institute space or Talaraich space). For studies where multiple independent patient samples were compared with HC, we included the appropriate coordinates as separate datasets. To avoid sample overlaps: (1) in the case of longitudinal studies, we included only the pretreatment data; (2) in the case of multiple studies using the same patient group, we included only the largest sample.

### Recorded variables

We recorded the following variables for each included study: sample size, gender and mean age of subjects; gestational age, birth weight, and the scores of Wechsler intelligence scales (either the WISC-R or the WAIS-III depending on subjects’ age); and the method used to correct whole-brain results for multiple comparisons.

### Standard meta-analyses of GMV and WMV abnormalities

We conducted separate voxel-based meta-analyses of GMV and WMV abnormalities using the SDM software package (http://www.sdmproject.com). We re-created maps of the effect size of group differences in GMV and WMV by using the peak coordinates. Both negative and positive coordinates were reconstructed in the same map to prevent a particular voxel appearing to be significant in opposite directions. The re-creation is based on converting the peak t value to Hedges’ effect size and then applying a non-normalized Gaussian kernel to the voxels close to the peak. Importantly, we also included negative studies. We used a threshold of p = 0.005 with peak Z >1 and a cluster extent of >50 voxels[24].

### Sensitivity analysis

To test the replicability of the results, we used a systematic whole-brain voxel-based jackknife sensitivity analysis. We repeated the main analysis 8 times for GMV and 6 times for WMV, discarding a different study each time. If a previously significant brain region remained significant in all or most of the combinations of the studies, it could be concluded that this finding was highly replicable.

### Heterogeneity and publication bias analysis

We examined the statistical (between-studies) heterogeneity of individual clusters using a random-effects model with Q statistics (χ2 distribution converted to z values) and tested with a permutation approach. The potential publication bias was formally assessed with Egger tests. We created funnel plots of the peaks of the main findings in order to discard gross abnormalities.

### Meta-regression analysis

We explored the following variables by meta-regression: the percentage of males, mean age of subjects at testing, gestational age, birth weight, and the scores of Wechsler intelligence scales (full IQ). In order to minimize the detection of spurious relationships, we decreased the probability threshold to 0.0005 and required abnormalities to be detected both in the slope and in one of the extremes of the regression, and discarded findings in regions other than those detected in the main analyses. Furthermore, we inspected regression plots to discard fittings driven by too few studies[24].

## Results

### Included studies and sample characteristics

**Fig 1:**
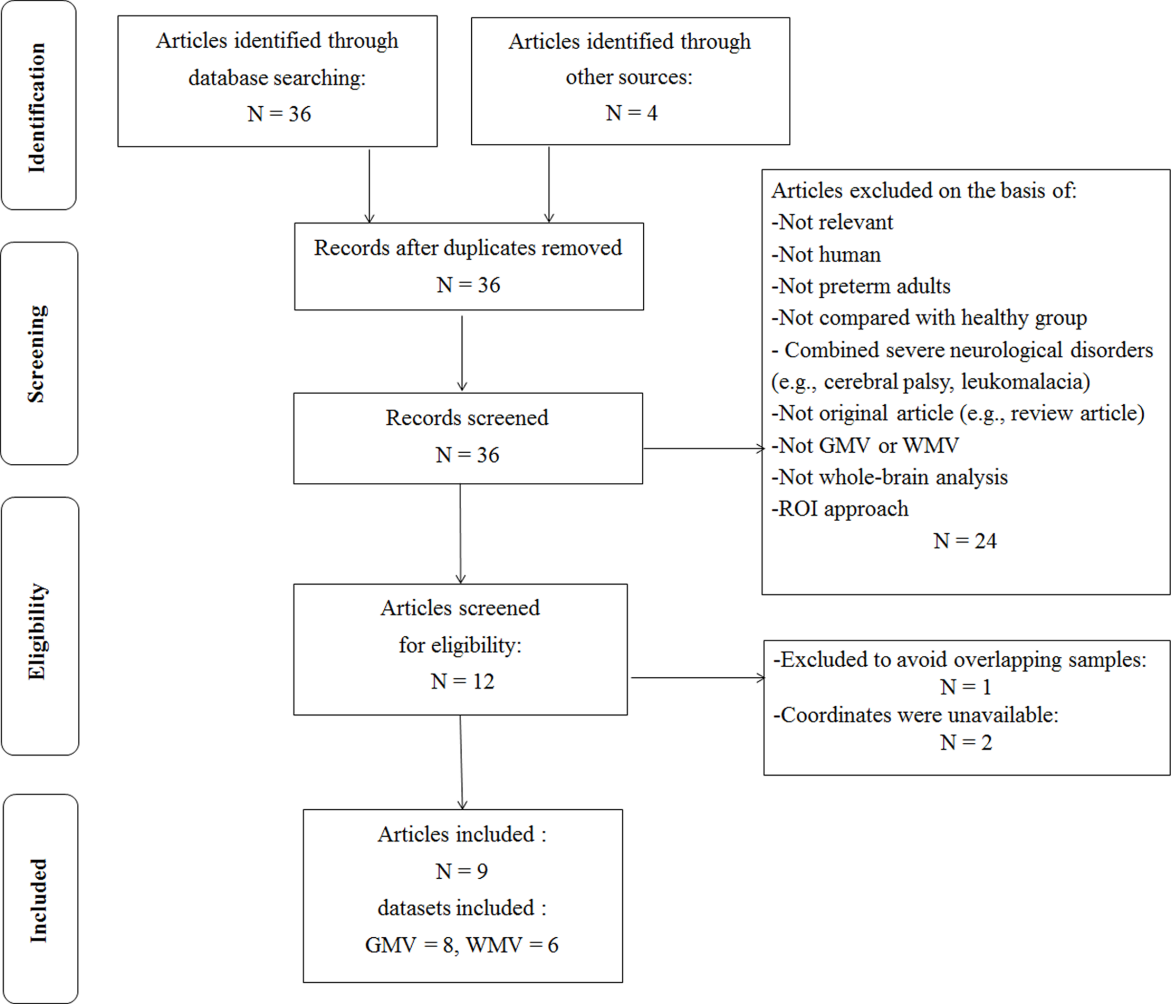
Search strategy used. Study selection was done according to “Preferred reporting items for systematic reviews and meta-analysis” (PRISMA) guidelines. Abbreviation: GMV, gray matter volume; ROI, region of interest; WMV, white matter volume.

The search strategy identified 36 studies after the duplicates were removed. Based on the stated inclusion criteria, a total of 9 articles[14–19, 25–28] (8 datasets of GMV from 7 articles, and 6 datasets of WMV from 5 articles) were ultimately included, with 633 PBA (314 males and 319 females; mean age 15.2 years) and 485 HC (258 males and 227 females; mean age 15.1 years). Two studies used overlapping samples[15, 28]: Nosarti’s study reported WMV findings and GMV findings, and Scott’s study only had GMV findings. After email discussion with these authors we selected Scott’s GMV findings and Nosarti’s WMV findings. One study compared male and female samples with HC separately, so the appropriate coordinates were included as two separate datasets[14]. Ultimately, we included 8 datasets of GMV and 6 datasets of WMV in the meta-analysis. Fig 1 shows a flow diagram of the identification and attrition of the studies. Clinical and demographic data are summarized in Table 1.

**Table 1:** Demographic and clinical characteristics of subjects in the 9 voxel-based morphometry studies included in the meta-analysis.

Study	Gray matter	White matter	Subject,n(male,n)		Age at testing (years)		Gestational age (weeks)	Birth weight (g)	Full IQ (score)	Statistical threshold
			PBA	HC	PBA	HC				
Gimenz(2006)[25]	N	Y	50(24)	50(22)	14.5	14.5	29.9	1327	103.0	corrected
Kesler(2008)[14]	Y	Y	17(17)	10(10)	12.2	12.2	28.3	945	N.A.	FWE corrected
Kesler(2008)[14]	Y	Y	12(0)	12(0)	12.3	12.2	28.7	1000	N.A.	FWE corrected
Nosarti(2008)[15]	N	Y	207(115)	104(59)	15.2	15.2	29.1	1276	N.A.	FWE corrected
Soria(2009)[17]	Y	Y	20(11)	22(14)	9.3	9.3	32.5	1754	105.8	corrected
Nagy(2009)[29]	Y	N	74(23)	69(16)	14.9	14.3	28.5	1070	N.A.	corrected
Scott(2011)[28]	Y	N	207(115)	104(59)	15.2	15.0	29.1	1276	N.A.	FWE corrected
Nosarti(2014)[18]	Y	Y	68(32)	43(13)	20.2	19.3	28.9	1225	96.2	FWE corrected
Meng(2014)[19]	Y	N	85(47)	69(44)	26.45	26.35	30.7	1356	95.3	FWE corrected
Lean(2017)[16]	Y	N	100(50)	106(57)	12.1	12.2	27.9	1063.5	N.A.	FWE corrected

Abbreviation: FWE = familywise error correction; HC = healthy controls; IQ = Intelligence Quotient; N.A. = not available; PBA = preterm-born adolescents.

### Changes in regional gray matter volume

**Fig 2:**
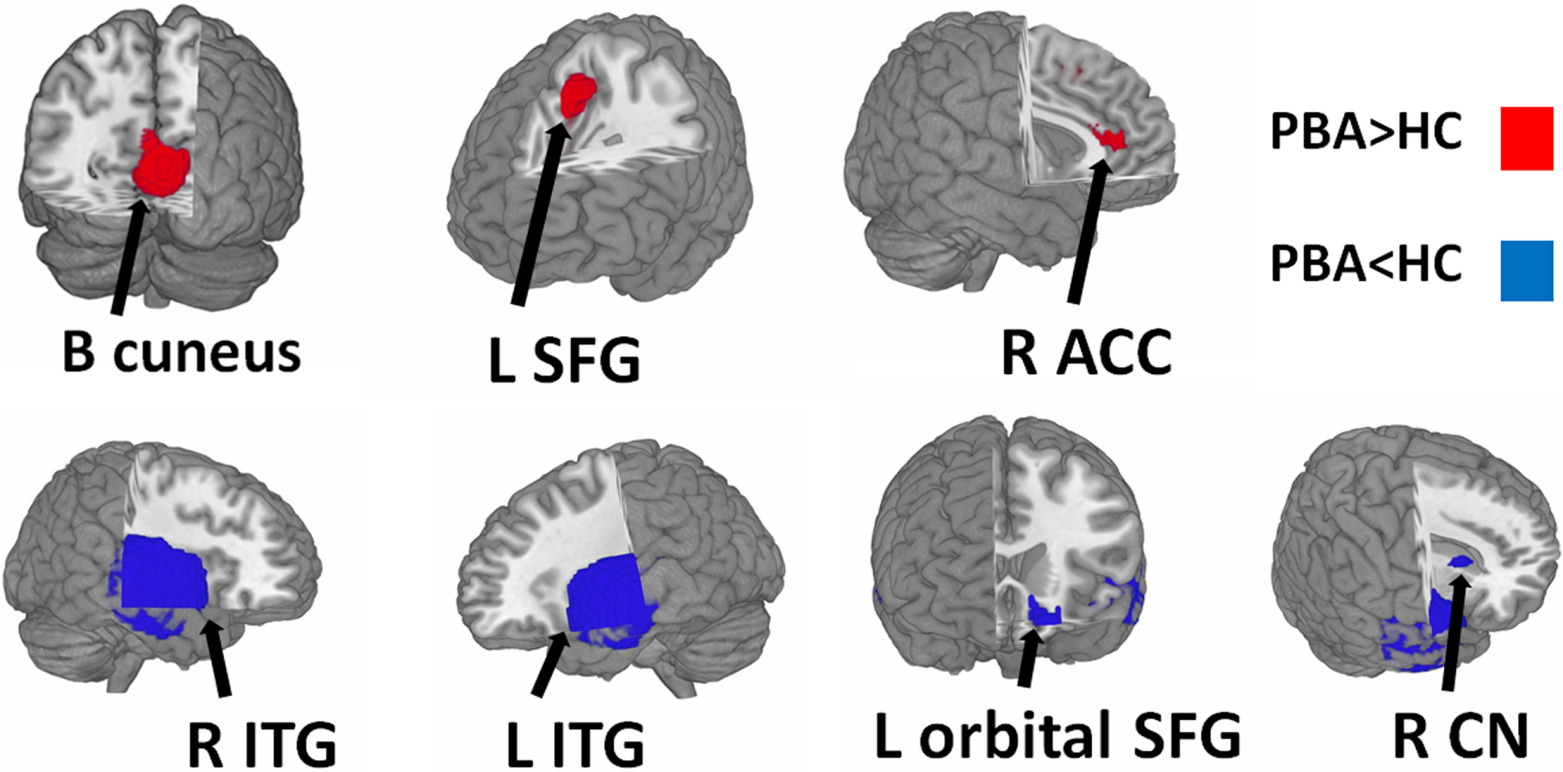
Regions showing increased (red) and decreased (blue) GMV in PBA compared with healthy controls. Abbreviation: ACC, anterior cingulate cortex; CN, caudate nucleus; ITG, inferior temporal gyrus; L, left; R, right; SFG, superior frontal gyrus.

The main GMV meta-analysis, a group comparison of PBA with HC across the 8 datasets, showed increased GMV relative to HC in the left cuneus cortex, the left medial superior frontal gyrus (mSFG), and the right dorsal anterior cingulate cortex (dACC); and decreased GMV relative to HC in the bilateral inferior temporal gyrus (ITG) extending to the middle and superior temporal gyrus, the orbital part of left superior frontal gyrus (SFG), and the right caudate nucleus (CN) (Table 2, Fig 2).

**Table 2:** Regional differences in gray matter and white matter volume between PBA and HC subjects identified by the present meta-analyses (voxelwise p<0.005 and FWHM 20 mm).

Region	Talairach coordinates			SDM z score		P, uncorrected	Voxel, n	Cluster breakdown(voxel, n)
	x	y	z					
**Pooled meta-analysis of all eligible studies of gray matter**								
**PBA>HC**								
Left cuneus cortex, BA 18	0	-88	22		1343	0.001230597	388	Left cuneus cortex, BA 18(311)
								Right cuneus cortex, BA 18(77)
Left superior frontal gyrus, medial, BA 8	-4	26	44		1.554	0.000495434	376	Left superior frontal gyrus, medial, BA 8(376)
Right anterior cingulate, BA 32	12	40	8		1.363	0.001138210	151	Right anterior cingulate, BA 32(98)
								Right median cingulate (30)
								Left anterior cingulate, BA 24(23)
**PBA<HC**								
Right inferior temporal gyrus, BA 20	48	-2	-14		-4.061	0.000006199	5504	Right inferior temporal gyrus, BA 20,21(1721)
								Right middle temporal gyrus, BA 20,21,22(1596)
								Right superior temporal gyrus, BA 21,38,40(1132)
								Right insula, BA 48(1055)
Left inferior temporal gyrus, BA 20	-46	-8	-16		-3.967	0.000008523	4769	Left inferior temporal gyrus, BA 20,21(1567)
								Left middle temporal gyrus, BA 21,48(1259)
								Left superior temporal gyrus, BA 22,48(1098)
								Left insula, BA 48(845)
Left superior frontal gyrus, orbital part, BA 11	-15	29	-20		-2.198	0.003046155	182	Left superior frontal gyrus, orbital part, BA 11(182)
Right caudate nucleus	10	14	4		-2.197	0.003058553	56	Right caudate nucleus(56)
**Pooled meta-analysis of all eligible studies of white matter**								
**PBA>HC**								
Right fusiform gyrus, BA 37	38	-48	-16		2.934	0.000010312	615	Right fusiform gyrus, BA 18,30, 37(615)
Right precuneus, BA 30	4	-52	14		2.920	0.000015497	103	Right precuneus, BA 29,30(103)
**PBA<HC**								
Left inferior temporal gyrus, BA 20	-42	-10	-16		-5.404	~0	1739	Left inferior temporal gyrus, BA 20,21(711)
								Left middle temporal gyrus, BA 20,21(529)
								Left insula, BA 48(313)
								Left superior temporal gyrus, BA 22,38,48(186)
Right inferior temporal gyrus, BA 20	46	-2	-26		-4.278	0.000030994	1447	Right inferior temporal gyrus, BA 20(672)
								Right middle temporal gyrus, BA 21(441)
								Right superior temporal gyrus, BA 21,22,38(334)
Left cortico-spinal projections	-6	-22	-8		-2.960	0.000495434	259	Left anterior thalamic projections(259)
Right inferior frontal gyrus	48	8	10		-3.599	0.000108361	255	Right inferior frontal gyrus (255)
Right cortico-spinal projections	8	-24	-4		-2.904	0.000541866	117	Left anterior thalamic projections(117)

Abbreviation: BA = Brodmann area; FWHM = full width at half maximum; HC = healthy control; PBA = preterm-born adolescents; SDM = Seed-based d Mapping.

Whole-brain jack-knife sensitivity analysis showed that increased GMV in the left cuneus cortex, left mSFG, right dACC and decreased GMV in the bilateral ITG and the orbital part of left SFG were highly replicable, being preserved throughout all 8 combinations of datasets. Decreased GMV in the right CN remained significant in all but two combinations[19, 27].

The regions with altered GMV did not showed significant statistical heterogeneity between studies (p > 0.005).

In analysis of publication bias, the Egger test was nonsignificant in the left cuneus cortex (p = 0.60), left mSFG (p = 0.60), right dACC (p = 0.85), right ITG (p = 0.23), left ITG (p = 0.39), the orbital part of left SFG (p = 0.81), and right CN (p = 0.85) (S1 Fig). Funnel plots demonstrated that the main findings were driven by at least 6 datasets (S1 Fig).

**Fig 3:**
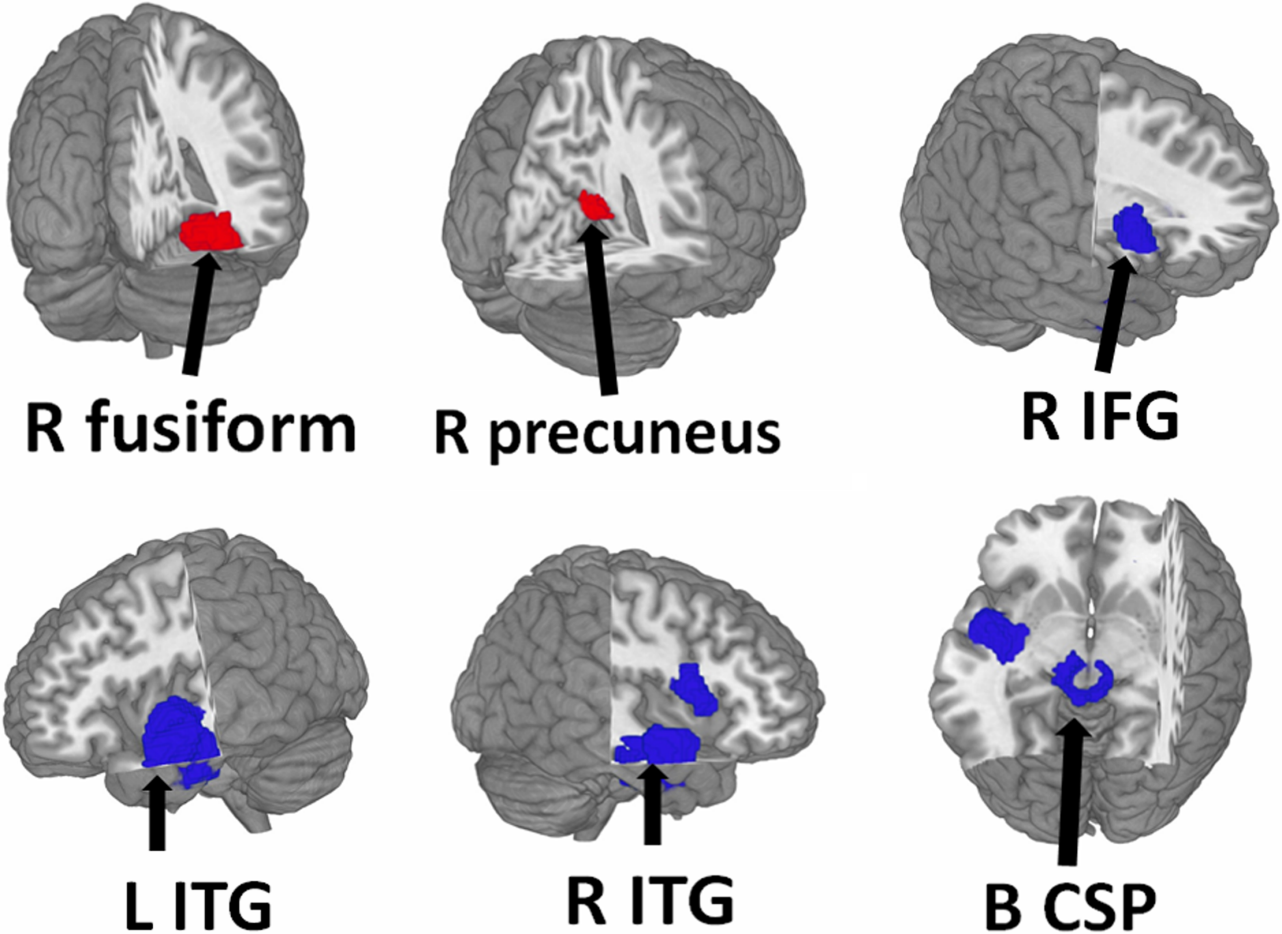
Regions showing increased (red) and decreased (blue) WMV in PBA compared with healthy controls. Abbreviation: B, bilateral; CSP, cortico-spinal projections; IFG, inferior frontal gyrus; L, left; R, right.

As shown in Fig 3, the percentage of male PBA was negatively correlated with decreased GMV in the left ITG (r = -0.407, permutation-derived p < 0.0001) and the right ITG (r = -0.554, permutation-derived p < 0.0001) in meta-regression analyses. The age at testing, gestational age, birth weight and full IQ were not linearly correlated with any GMV findings in PBA.

### Changes in regional white matter volume

**Fig 4:**
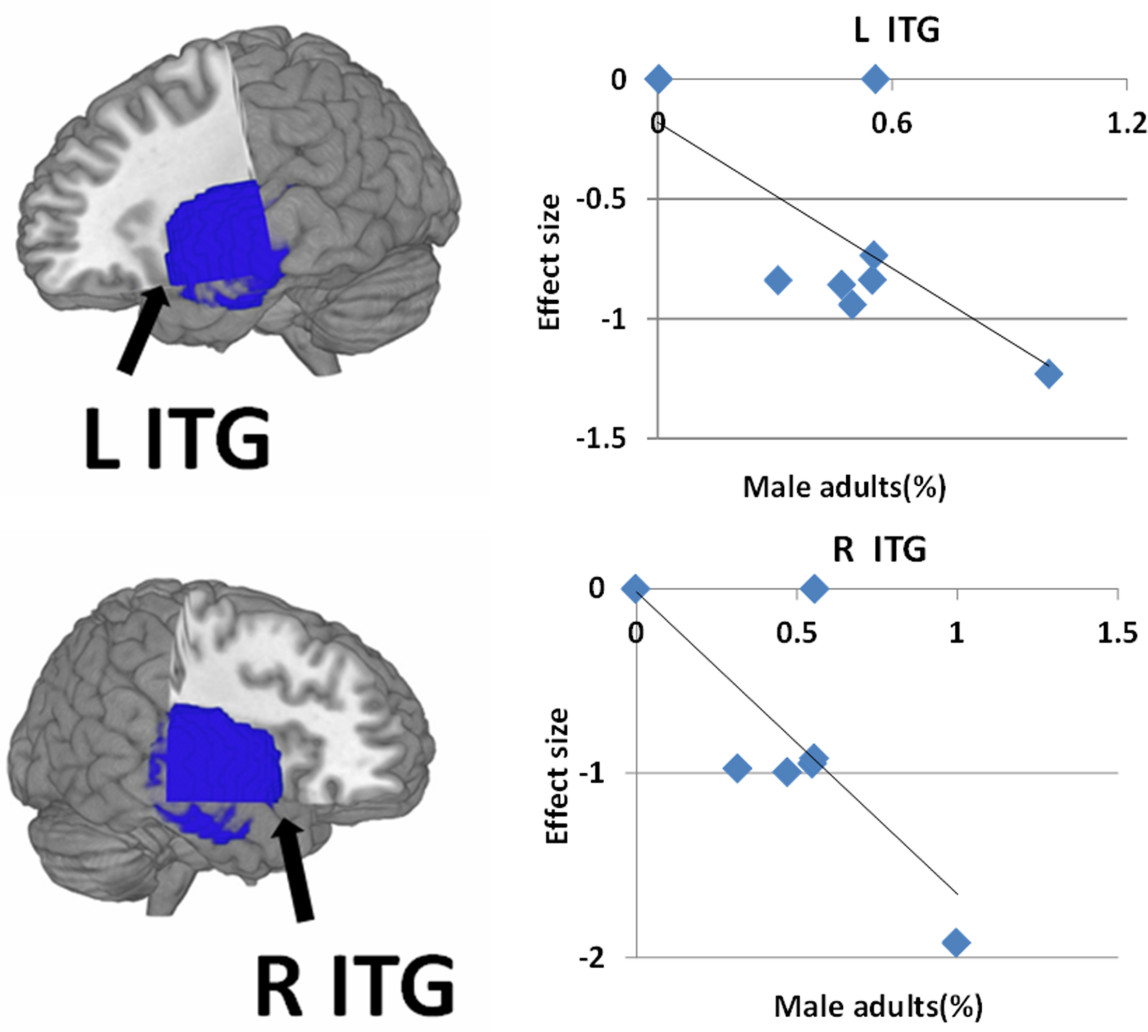
Meta-regression results show that the percentage of male patients was negatively correlated with gray matter in the bilateral temporal lobe. In the graphs, the effect sizes needed to create this plot have been extracted from the peak of maximum slope significance, and each study is represented as a dot. The regression line (meta-regression signed differential mapping slope) is shown. Abbreviation: ITG, inferior temporal gyrus; L, left; R, right.

The main WMV meta-analysis, a group comparison of PBA with HC across the 6 datasets, showed increased WMV relative to HC in the right fusiform gyrus and right precuneus; and decreased WMV relative to HC in the bilateral ITG, the bilateral cortico-spinal projections, and the right inferior frontal gyrus (IFG) in PBA (Table 2, Fig 4).

Whole-brain jackknife sensitivity analysis showed that increased WMV in the right fusiform gyrus, and right precuneus, and also decreased WMV in bilateral ITG, bilateral cortico-spinal projections, and right IFG, were all highly replicable, being preserved throughout all 6 combinations of datasets.

The regions with altered WMV did not show significant statistical heterogeneity between studies (p > 0.005).

In analysis of publication bias, the Egger test was nonsignificant in the right fusiform gyrus (p = 0.06), right precuneus (p = 0.06), bilateral cortico-spinal projections (p = 0.07 / 0.08), and the right IFG (p = 0.14) (S1 Fig). Funnel plots showed that the main findings were driven by at least 6 datasets (S1 Fig).

Meta-regression analyses revealed that the changes of WMV-related brain abnormal in PBA were not associated with the percentage of male PBA, age at testing, gestational age and birth weight and full IQ, at least linearly.

## Discussion

To our knowledge this is the first voxel-wise meta-analysis of VBM studies in PBA examining GMV and WMV abnormalities. The PBA group, compared with HC, showed significantly and robustly decreased GMV and WMV in the bilateral ITG extending to the middle and superior temporal gyrus. PBA also showed increased GMV in the left cuneus cortex, the left mSFG, and the right dACC; decreased GMV in the orbital part of left SFG, and the right CN; increased WMV in the right fusiform gyrus and right precuneus; and decreased WMV in the bilateral cortico-spinal projections and the right IFG. Meta-regression analyses revealed that the percentage of male PBA was negatively associated with decreased GMV in the bilateral ITG.

Next we discuss what these abnormalities might tell us about pathophysiology.

### Inferior temporal gyrus, medial prefrontal cortex and precuneus: The default mode network

The most prominent findings were decreased GMV and WMV in the bilateral ITG, extending to the middle and superior temporal gyrus; almost the entire temporal lobe is affected. Volume reductions in the temporal gyrus were previously reported in PBA[17]. It is known that brain maturation starts in the central area, proceeds toward the parieto-occipital cortex, and only then reaches the temporal lobe. Late development of these regions might make these structures more vulnerable to the influence of environmental factors during childhood[29]. As can be seen in the SDM maps, the GMV abnormalities get very close to the WMV abnormalities in the bilateral temporal lobe (compare Figs 2 and 3). One possible mechanism is exaggeration of synaptic pruning (‘hyperpruning’), a process which normally refines interneuron function and connectivity from adolescence through early adulthood[30]. Alternatively, abnormal gray matter architecture may have effects on both distant and adjacent brain regions, which fail to receive input from the damaged cortex [31].

Dysfunction of the ITG is important in cognitive impairment[32], of which PBA are at significant risk[33]. The STG is important in the development of linguistic abilities such as reading and spelling[34], and language difficulties are common in children born prematurely[7]. Thus the decreased GMV and WMV we found in the bilateral temporal lobe may be causally important in cognitive impairment and language difficulties in PBA.

The PBA group also showed increased GMV in the left mSFG, a part of the medial prefrontal cortex (mPFC) which is implicated in spelling and language ability [28], intellectual ability[35], cognitive outcome and emotion regulation[36]. We also found increased WMV in the precuneus.

The ITG, mPFC and precuneus are critical components of the default mode network (DMN), which functions in emotion regulation[37], language comprehension and cognition[38]. The period of 32–40 weeks gestational age is an important developmental epoch for the DMN[39], and this may underlie damage to the DMN system in PBA. Abnormalities in the DMN have also been identified in almost every major psychiatric disorder including anxiety, depression, autism and attention deficit hyperactivity disorder (ADHD)[38, 40], which might explain why these are all common in PBA. DMN dysfunction may therefore be the main pathophysiological mechanism in PBA.

### Cuneus and fusiform gyrus: The visual recognition network

Compared with HC, PBA showed increased GMV in the cuneus and increased WMV in the fusiform gyrus, which are both parts of the visual recognition network. Increases in both GMV and WMV may reflect an abnormal or delayed pruning program, thus reflecting both destructive and adaptive developmental processes [15]. The visual recognition network is important for visual perception and related functions such as learning, memory and interactions with the visual world[41]. The cuneus is part of the occipital lobe. Rapid occipital development may make this region more vulnerable to premature birth, which might underlie the visual impairment in PBA[42]. The fusiform gyrus is important implications in the development of social skills[43], and this abnormality may be causally related to the ‘socially immaturity’ of PBA. Thus alterations in the visual recognition network may be closely related to both visual impairment and social immaturity in PBA.

### Orbital frontal cortex and dorsal anterior cingulate cortex: The salience network

The PBA group showed decreased GMV in the orbital part of left SFG corresponding to the left medial orbital frontal cortex (OFC). Decreased GMV in OFC has been previously observed in VPT samples[15], and moreover, has been found to correlate with intellectual ability in PBA[35]. There are other reports relating social dysfunction in PBA to structural abnormalities in OFC [13], because of its importance in social regulation, social cognition and theory of mind [22].

The PBA showed increased GMV in the right dACC. The dACC is thought to play a role in regulating attention and monitoring task performance [44]. This may why PBA are at increased risk of attention problems, being twice as likely as full-term children to be diagnosed with attention deficit hyperactivity disorder (ADHD) [45]. Bora et al. found that reduced cerebral tissue volumes in OFC at term were associated with attention/hyperactivity problems in PBA from age 4 to 9 years[46].

The OFC and dACC are core nodes in the salience network (SN), which is involved in cognitive and behavioral phenomena related to decision-making and cognitive control [47]. It may therefore be that these GMV alterations in the OFC and dACC are causally related to the impairments of cognition, motor control, attention and social function in PBA [48, 49].

### Gender effects

Meta-regression analyses showed that the percentage of male PBA was negatively associated with decreased GMV in the bilateral ITG: that is, male PBA tend to have a smaller temporal lobe GMV. This may be because delayed myelination is reportedly more frequent in male than female PBA[50]. Myelination is critical for normal neurodevelopment and may be adversely affected by preterm birth [50]. Male PBA are more likely than females to have moderate to severe cerebral palsy, and this has been related to poor general neuromotor behavior outcome at age 7 years [51]. Sex differences are also seen in cognitive aspects: male PBAs had lower cognitive and language scores than females [52]. It may therefore be that the sex differences of GMV in bilateral ITG contribute to differences in cognitive aspects of PBA.

### Limitations and conclusions

This study has several limitations. First, the relatively small number of eligible studies, some with rather small and selective samples of PBA, limits the generalizability of the results, especially in the meta-regression. Second, peak-based meta-analyses are based on summarized data (i.e. coordinates from published studies) rather than raw statistical brain maps, and this may result in less accurate results[53]. Third, there are no prospective randomized controlled studies of the long-term effects of preterm birth on brain structure. Fourth, a number of studies provided limited information about clinical characteristics and social background.

In summary, to our knowledge this is the first voxel-wise meta-analysis of VBM studies in PBA. PBA show widespread GMV and WMV alterations mainly in the default mode network (ITG, mPFC and precuneus), visual recognition network (cuneus and fusiform gyrus), and salience network (dACC). These changes may be causally associated with socialization difficulties, educational underachievement, language impairment, cognitive impairment, and behavioral problems in PBA. Meta-regression suggests that male PBA tend to have smaller temporal lobe GMV, which may reflect the structural underpinnings of the sex differences observed in cognitive aspects of PBA. Future studies will benefit from the use of a longitudinal approach to investigate dynamic brain structure changes and the influence of external and internal factors on brain development in PBA.

## Supporting information

S1 Fig Results of the funnel plot analysis. (DOCX)

S1 Table PRISMA checklist. (DOC)
