# Supplementary material for: Brain gray and white matter abnormalities in preterm-born adolescents: A meta-analysis of voxel-based morphometry studies
Source: PLoS One. 2018 Oct 10;13(10):e0203498. doi: 10.1371/journal.pone.0203498 (PMC6179190; doi:10.1371/journal.pone.0203498)
Supplement: S1 Fig — (DOCX) [file pone.0203498.s001.docx]

**Supplementary material**

**Figure S1**. Results of the funnel plot analysis

| **Gray matter volume (GMV)** | |
| --- | --- |
| Left cuneus cortex, BA 18（0，-88，22） | Egger test: Bias: -0.57, t: -0.55, df: 6, p: 0.604  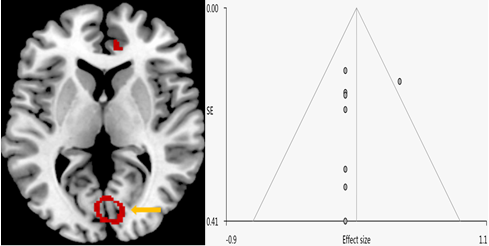  Funnel plot showed that seven studies contributed to this cluster, and one to the other clusters. |
| Left superior frontal gyrus, medial, BA 8（-4，26，55） | Egger test: Bias: -0.51, t: -0.55, df: 6, p: 0.600  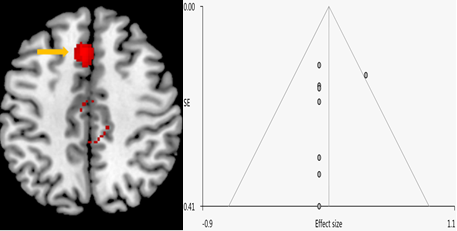  Funnel plot showed that seven studies contributed to this cluster, and one to the other clusters. |
| Right anterior cingulate, BA 32（12，44，8） | Egger test: Bias: 0.22, t: 0.20, df: 6, p: 0.850  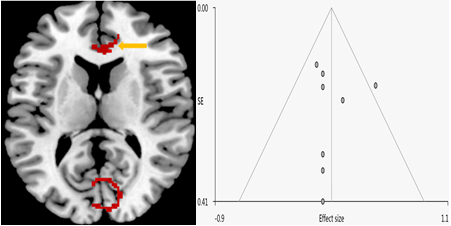  Funnel plot showed that seven studies contributed to this cluster, and one to the other clusters. |
| Right inferior temporal gyrus, BA 20（48，-2，-14） | Egger test: Bias: -3.19, t: -1.32, df: 6, p: 0.234  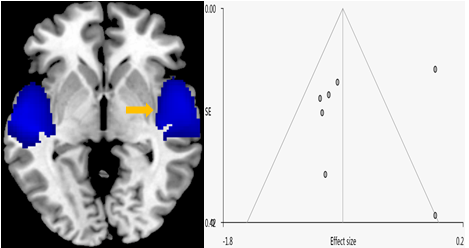  Funnel plot showed that six studies contributed to this cluster, and two to the other clusters. |
| Left inferior temporal gyrus, BA 20（-46，-8，-16） | Egger test: Bias: -2.16, t: -0.93, df: 6, p: 0.386  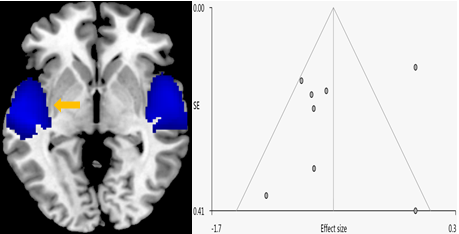  Funnel plot showed that seven studies contributed to this cluster, and one to the other clusters. |
| Left superior frontal gyrus, orbital part, BA 11（-15，29，-20） | Egger test: Bias: -0.38, t: -0.25, df: 6, p: 0.808  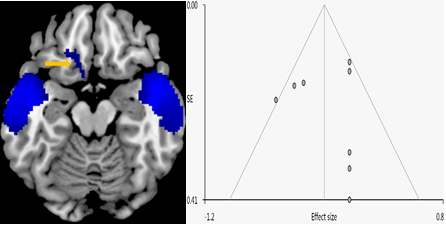  Funnel plot showed that eight studies contributed to this cluster. |
| Right caudate nucleus（10，14，4） | Egger test: Bias: -0.26, t: -0.20, df: 6, p: 0.849  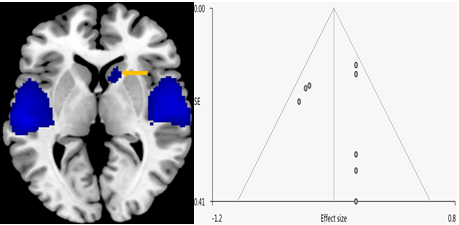  Funnel plot showed that eight studies contributed to this cluster. |
| **White matter volume (WMV)** | |
| Right fusiform gyrus, BA 37(38,-48,-16) | Egger test: Bias: -1.83, t: -2.50, df: 4, p: 0.067  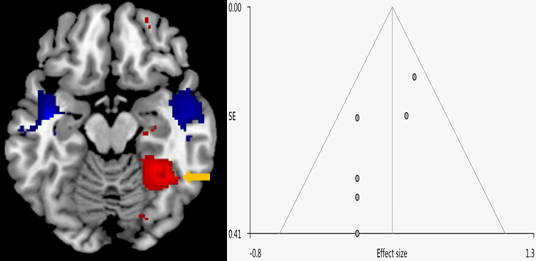  Funnel plot showed that seven studies contributed to this cluster. |
| Right precuneus, BA 30(4,-52,14) | Egger test: Bias: -1.83, t: -2.62, df: 4, p: 0.059  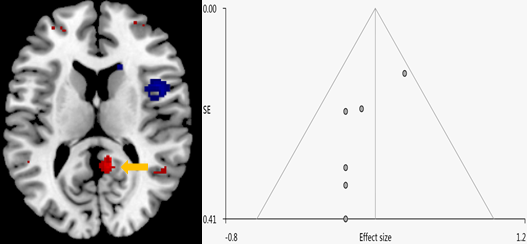  Funnel plot showed that seven studies contributed to this cluster. |
| Left inferior temporal gyrus, BA 20(-42,-10,-16) | Egger test: Bias: 2.02, t: 4.35, df: 4, p: 0.012  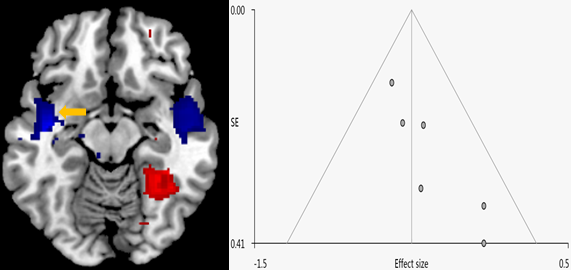  Funnel plot showed that seven studies contributed to this cluster. |
| Right inferior temporal gyrus, BA 20(46,-2,-26) | Egger test: Bias: 1.85, t: 3.20, df: 4, p: 0.033  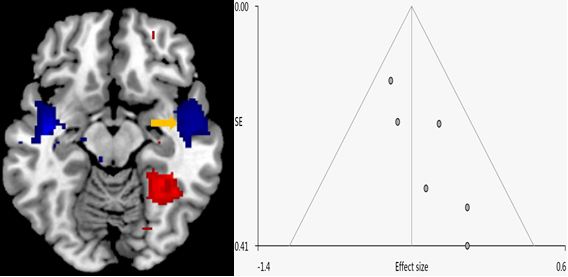  Funnel plot showed that seven studies contributed to this cluster. |
| Left cortico-spinal projections(-6,-22,-8) | Egger test: Bias: 1.82, t: 2.41, df: 4, p: 0.074  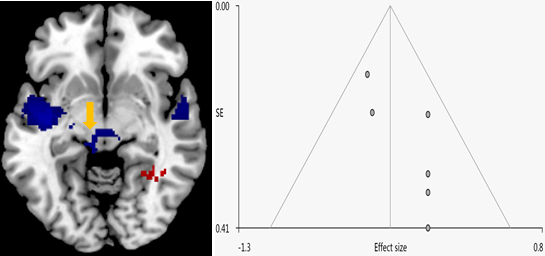  Funnel plot showed that seven studies contributed to this cluster. |
| Right inferior frontal gyrus (48,8,10) | Egger test: Bias: 1.73, t: 2.53, df: 4, p: 0.065  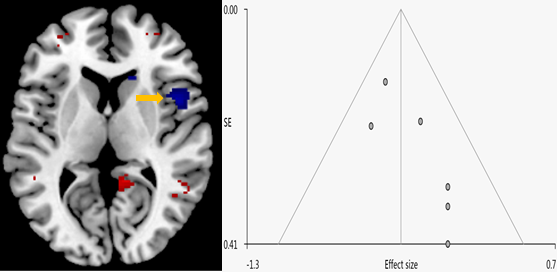  Funnel plot showed that seven studies contributed to this cluster. |
| Right cortico-spinal projections(8,-24,-4) | Egger test: Bias: 1.60, t: 1.83, df: 4, p: 0.141  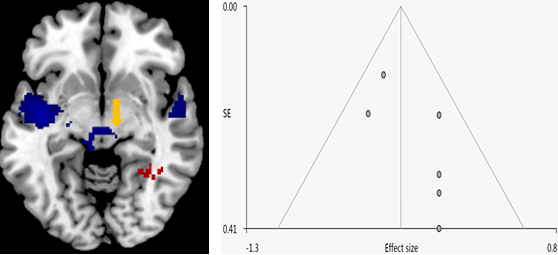  Funnel plot showed that seven studies contributed to this cluster. |
